# Why choose the treatment with cisplatin for BRCA1 breast cancers patients?

**DOI:** 10.1186/1897-4287-10-S1-A4

**Published:** 2012-01-12

**Authors:** T Byrski, T Huzarski, E Marczyk, P Blecharz, J Gronwald, O Ashuryk, C Cybulski, T Dębniak, D Zuziak, D Godlewski, J Kladny, J Lubinski, SA Narod

**Affiliations:** 1 Pomeranian Medical University, Szczecin, Poland; 2 Oncology Institute, Kraków, Poland; 3 Regional Oncology Center, Bielsko-Biała, Poland; 4 Center for Epidemiology and Prevention, Poznań, Poland; 5 Women’s College Research Institute, Toronto, Ontario, Canada

## Purpose

To identify host and tumor factors which predict a complete pathologic response (pCR) after neoadjuvant treatment with cisplatin chemotherapy in women with breast cancer and a BRCA1 mutation.

## Patients and methods

50 women with breast cancer and a BRCA1 mutation, who presented with stage I to III breast cancer between December 2006 and April 2010 were enrolled and treated with cisplatin 75 mg/m^2^ every three weeks for four cycles, followed by mastectomy and conventional chemotherapy. Eight patients had prior chemotherapy for a previous cancer diagnosis. Three patients received prior chemotherapy for their current cancer diagnosis but received protocol therapy and thus were followed for toxicity and evaluated for response. Information was collected on clinical stage, grade, hormone receptor status and Her2neu (HER2) status prior to treatment. Pathologic complete response was determined by review of surgical specimens.

## Results

47 patients were enrolled in the study, including 39 patients for whom this was the first primary breast cancer and who had no previous chemotherapy and 8 patients who had previously received chemotherapy. A pathologic complete response (pCR) was observed in 34 patients (68%) including 82% of women who had no previous chemotherapy and 18% of women who had previously received chemotherapy.

## Conclusions

Platinum-based chemotherapy is effective in a high proportion of patients with BRCA1-associated breast cancer, but may be less effective in women who have been previously been treated with another form of chemotherapy.

